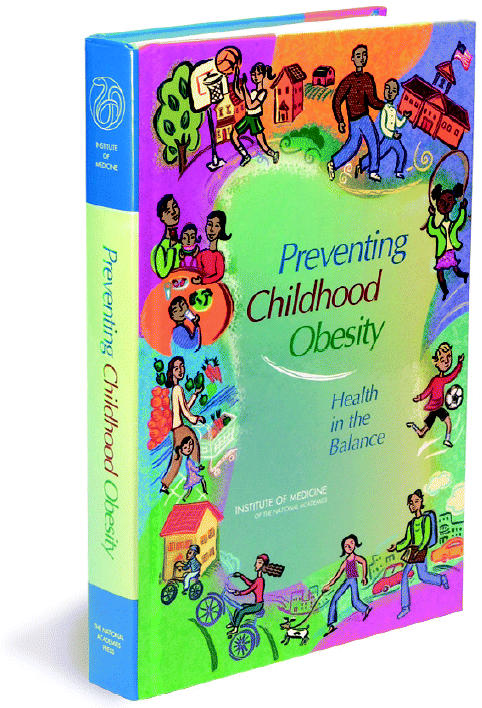# Preventing Childhood Obesity: Health in the Balance

**Published:** 2005-10

**Authors:** Jeffrey B. Schwimmer

**Affiliations:** Jeffrey B. Schwimmer is assistant professor of pediatrics in the Devision of Gastroenterology, Hepatology, and Nutrition; Department of Pediatrics; University of California, San Diego. He is the director of Weight and Wellness at the Children's Hospital and Health Center.

By Jeffrey P. Koplan, Catharyn T. Liverman, and Vivica I. Kraak

Washington, DC:National Academies Press, 2005. 414 pp. ISBN: 0-309-09315-5, $44.95 cloth

On a local daily level, what do 9 million obese children look like? In the typical classroom in America today there are four or five obese students. At an urban high school, set in a low-income neighborhood, serving an ethnic minority population, one might find nearly half the teens overweight or obese. Pediatric clinics are increasingly confronted with children and adolescents suffering from obesity-related health problems. Because of the global complexity of factors influencing the development of obesity and the speed at which childhood obesity has become commonplace, our society is unprepared to adequately address the crisis.

*Preventing Childhood Obesity: Health in the Balance* is a book-length report based on a 24-month study by a national committee of thought leaders from across the nation in response to the epidemic of childhood obesity. Three years ago, directed by the U.S. Congress and the Centers for Disease Control and Prevention, the Institute of Medicine (IOM) charged a committee to develop a prevention-focused plan to decrease the prevalence of obesity in children and youth in the United States.

The book presents a broad range of data to build the case for launching a large-scale effort befitting a national public health priority. Although the prevalence of obesity in America’s youth and its health and psychosocial consequences are well documented, limited data are available on how to effectively reverse the crisis. Like an exploring cartographer, to prevent childhood obesity we must discover and record the routes at the same time we are traversing them; to wait until they are delineated is not an option. Such a belief led the committee to recommend actions “based on the best available evidence—as opposed to waiting for the possible evidence.” They produced 10 recommendations on national priority, industry, nutrition labeling, advertising and marketing, multimedia and public relations, community programs, built environment, health care, schools, and home.

Many of these areas are fraught with controversy in the interface of government, industry, and schools. Even the term used—obesity—carries a controversial definition; the word “obese” is loaded with negative connotations. However, in the context of health, the word is not interchangeable with “overweight.” This latter term trivializes the importance of the problem. The committee took a major step forward by justifying and adopting a definition for pediatric “obesity” rather than “overweight”: For children 2–17 years of age, “obesity” is a body mass index ≥the 95th percentile for age and sex.

Of particular interest is the growing realization of the importance of the built environment in the societal decrease in physical activity and dietary quality. To address the relationship between the built environment and childhood obesity, the National Institute of Environmental Health Sciences convened national conferences in 2004 and 2005. The IOM committee recommended that local governments, private developers, and community groups expand opportunities for physical activity, including recreational facilities, parks, playgrounds, sidewalks, bike paths, routes for walking or bicycling to school, and safe streets and neighborhoods, especially for populations at high risk of childhood obesity.

The goal set by Healthy People 2010 is to reduce the proportion of obese children and adolescents to 5% by 2010. Given that 16% of children are obese, 5 years is simply not enough time to undo a problem of this magnitude. The committee states that “it will require a long-term commitment spanning many years and possibly decades because the epidemic has taken years to develop and will require persistent efforts and the investment of sustained resources to effectively ameliorate.” The IOM committee’s charge did not include the issue of treatment. Although preventive efforts hold promise for future generations, for millions of children and adolescents prevention comes too late. Therefore, secondary and tertiary prevention (treatment) must also become part of the national plan.

In 2001 the U.S. Surgeon General issued a call to action, sounding the alarm that obesity was a crisis. In 2005, this book provides a working document for taking action. It is incumbent on all of us to read this book and take action. Through our collective actions, this roadmap will become a living document that will grow and change over time. The committee stressed the need for evaluation of those efforts—to learn from both success and failure. The ultimate success will be to make the social norm one that prioritizes a healthy diet, activity level, and body weight for all children.

## Figures and Tables

**Figure:**